# 1803. Pediatric Antimicrobial Stewardship: Beyond the Core Elements

**DOI:** 10.1093/ofid/ofac492.1433

**Published:** 2022-12-15

**Authors:** Marisol Fernandez, Rachel Downey, Peter Gilbreath, Toni Wakefield, Hanh Keyburn, Joanna Schwartz, Melissa Cossey, Julia Sapozhnikov, Winnie Whitaker, Andrew Kienstra, Rebecca Floyed, Devika Pillay, Tiffany Robles, David Woerner, Marion Forbes

**Affiliations:** Dell Children's Medical Center of Central Texas, Dell Medical School at UT Austin, Austin, Texas; Dell Children's Medical Center of Central Texas, Austin, Texas; Dell Children's Medical Center; Dell Medical School at the University of Texas at Austin, Autsin, Texas; Dell Children's Medical Center; Dell Medical School at the University of Texas at Austin, Autsin, Texas; Dell Children's Medical Center; Dell Medical School at the University of Texas at Austin, Autsin, Texas; Dell Children's Medical Center, Dell Medical School at the University of Texas at Austin, Austin, Texas; Dell Children's Medical Center; Dell Medical School t the University of Texas at Austin, Autsin, Texas; Loyola University Medical Center, Maywood, Illinois; Dell Children's Medical Center; Dell Medical School at the University of Texas at Austin, Autsin, Texas; Dell Children's Medical Center; Dell Medical School at the University of Texas at Austin, Autsin, Texas; Dell Children's Medical Center; Dell Medical School at the University of Texas at Austin, Autsin, Texas; Dell Children's Medical Center; Dell Medical School at the University of Texas at Austin, Autsin, Texas; Dell Children's Medical Center; Dell Medical School at the University of Texas at Austin, Autsin, Texas; Dell Medical School at the University of Texas at Austin, Austin, Texas; Dell Children's Medical Center; Dell Medical School at the University of Texas at Austin, Autsin, Texas

## Abstract

**Background:**

Facility treatment guidelines for antibiotic choice and duration are a priority in the CDC Core Elements of hospital antimicrobial stewardship (AMS). Urinary tract infection (UTI) and community acquired pneumonia (CAP) are common pediatric diagnoses with potential for AMS impact in both inpatient and outpatient settings.

We describe a project at a free-standing children’s hospital that was implemented through a collaboration of multidisciplinary providers who participated in the national Value in Inpatient Pediatrics (VIP) program. One aim of this project is to evaluate baseline and improve antibiotic duration for CAP and UTI.

**Methods:**

This is a retrospective/prospective chart review study that compares baseline and post-intervention duration of antibiotic prescribing for UTI and CAP cases from July 2019-December 2021 based on data collected through the VIP program. Figure 1 describes the timeline of events and results. Patients included were identified by diagnosis code for CAP and UTI. Each case was randomized for inclusion and manually reviewed to ensure it met the diagnosis clinical definition. Cases with underlying chronic medical conditions were excluded from this analysis. Duration of antibiotics prescribed was compared for each group using Wilcoxon rank-sum testing.

**Figure 1.**
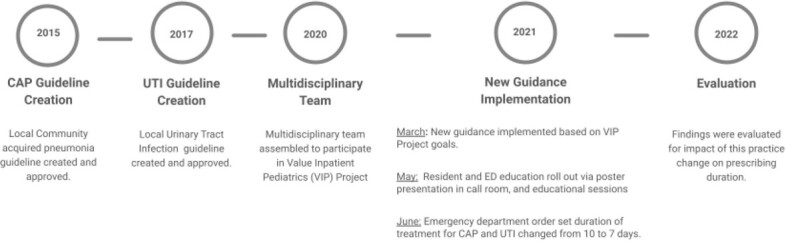
Timeline of guideline implementation and evaluation

**Results:**

Among the 351 patients included, 163 had a diagnosis of CAP (98 pre-, 65 post- implementation); 188 with UTI (121 pre-, 67 post- implementation). Post Implementation, there was a significant decrease in median duration of antibiotic prescribing from 10 to 7 days in both groups (p< 0.001).

**Figure 2.**
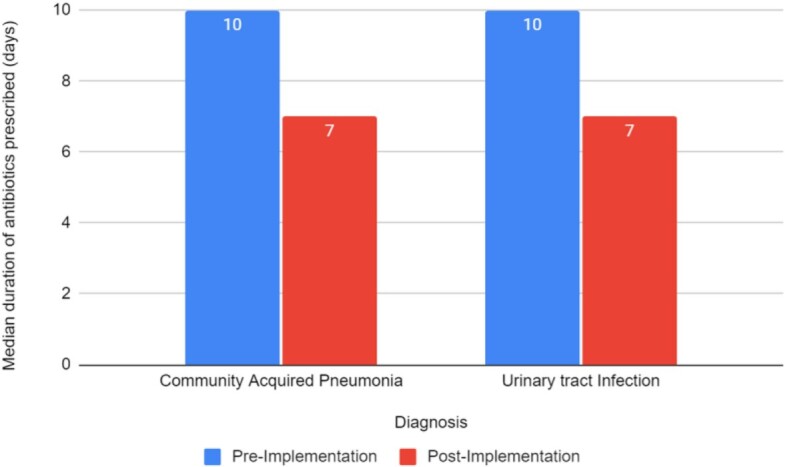
Median duration of antibiotics pre and post program implementation (p<0.001)

**Conclusion:**

Institutional implementation of guidelines is an important step in local AMS. Guideline implementation alone is not enough to ensure practice change and going beyond the CDC core elements has become important. In our case, participation in a national project with local multidisciplinary involvement was successful in improving duration of therapy for CAP and UTI not previously achieved by the local guidelines. Changes were made to the electronic medical record in the ED to ensure sustainability of this change.

**Disclosures:**

**All Authors**: No reported disclosures.

